# Monthly Summary

**Published:** 1890-04

**Authors:** 


### Monthly Summary.

SYNTHETIC CARBOLIC ACID.—Some experiments have been made by Dr. Ohlmuller, assistant in the German Imperial Health Department, to test the relative disinfecting power of "synthetic" carbolic acid, according to the *Pharmaceutische Zeitung*. The sample examined solidified at a moderate temperature to greasy needles that refracted light strongly, had a sp. gr. of 1.0681 and melted at 41° C. With the exception that the compound gave a faintly acid reaction with blue litmus paper it corresponded to all the requirements of the Pharmacopœa Germanica. The comparison was made with the ordinary official carbolic acid, as well as with two varieties of crude carbolic acid—oily and tarry—mixed with sulphuric acid, and the estimate of their relative power as disinfectants and antiseptics was based upon their antibacterial action. The conclusions arrived at were that there is a difference in the disinfecting power of the two kinds of carbolic acid, since the synthetic acid diminished the life activity of the bacteria experimented upon rather less than the older kind, but that the difference was so small that the two kinds might be considered to be practically of the same value. The disinfecting action of the mixture of crude carbolic acid and sulphuric acid was found, however, to be greater than that of either of the purer acids used alone.—*O. P. & D. Reporter.*

---

ERUPTION OF TEETH IN OLD AGE.—Mr. George F. Fisher reports in *The Lancet* the case of a lady, aged sixty-four, who asked him to lance her gums as they were very painful, having kept her awake for several nights. On examining the gums of the upper jaw he found a well-developed new canine tooth protruding, and the corresponding side full and painful, feeling to the touch distinctly as though a tooth were coming through. The patient had lost her incisor and canine teeth in the upper jaw some ten years before. A case is recently reported of a new-vender of 83 years who is now erupting a third set of teeth.

THE STOMACH-BRUSH.—A dental journal publishes the following, translated from the German: In 1713 there was published a pamphlet entitled, "A Complete Account of the most Useful Stomach Brush which is now to be had at the Brushmakers at the Old Court Sadler's Shop in Broad Street in Colln-on-the Spree." Many a one may have wished to be able once in a way to have his stomach thoroughly cleaned out, and this speculative brushmaker gave a practicable means to give effect to this wish. In the pamphlet there is a drawing of the stomach-brush; it resembles a pipe-cleaner, but, of course, is larger. The stalk is made of four wires twisted together, covered with thread, silk, or small ribbons; it is twenty-six inches long. The brush at the under end is two inches long and one and a half broad, and is made of goat's beard hair; but, when one has been accustomed to use it for three or four weeks, a horse-hair brush is substituted, this hair being somewhat stronger, and so the effect is better. The application of this most excellent brush is very simple. It is pressed through the throat down into the stomach, which, by drawing up and down of the brush, is cleaned; thereafter cold water or brandy is to be drunk, and the operation is repeated till the cleaning is perfect. The cure is to be repeated every morning. The author says: "At first you will find it rather troublesome to get the brush down, but when you put it in your mouth and on your palate, draw in breath and wind, and press it gently and gradually down, and, without any particular trouble, it will reach the stomach. After eight to fourteen days' practice, it will come as easily to you as eating or drinking." Of course, the daily application of the stomach-brush is the infallible remedy or preventive of all diseases that can be imagined. "Whoever uses this cure requires no other medicine, for it is good against all—cold, hot, and poisonous fevers, it gives a good appetite for eating, it is good against asthma, hemorrhage, headache, chest complaints, coughs, consumptions, apoplexy, toothache, sore eyes, dysentery, quinsy on the tongue, quinsy in the throat, ulcers, abscesses, cardialgy; it favors digestion, strengthens the heart, drives away pimples on the skin, is against choking in the stomach, etc., makes too fat and asthmatical and swollen-up people thin, and, on the other hand, makes meagre and thin people fat. The great effect, however, is produced only when the use of the brush is combined with that of an elixir. This is compounded of aloes, saffron, rhubarbona, lark-mushroom, wormseed, eugian, myrrh, theriac. After the stomach washing, forty to fifty drops of the elixir is to be taken in wine, and this preserves for twenty-four hours against all poison and pestilence."—*British Medical Journal.*

---

A SIMPLE REGULATING APPLIANCE.—*By Dr. Gillett.*—Another little thing I want to present: It was a very simple case in regulating, in which the upper central incisors in coming down caught between the lower ones in such a way as to stop their eruption. It was the case of a delicate little child, going to school, taking music lessons, and all that sort of thing, and it was desirable to make an especially simple appliance which could be worn without discomfort. The plan which I pursued for moving out the centrals was to cement to the first molars platinum caps, covering the grinding surfaces, and having soldered to their palatal aspects small tubes into which were slipped the ends of a piece of gold wire, gauge 22. This wire conformed to the outline of the palatal aspect of the arch, being supported in front by two little clips attached to hands about the central. Each end was threaded and carried a small set-nut which, when turned, impinged upon the tubes into which the wires were passed on the molar caps, and so carried the whole wire forward. The springing of the wire under this, kept a constant pressure upon the centrals doing the work in a few days. The point which I wish to come at, is a ready method of making the molar caps. This die is made of a fusible metal which may be run into a modeling compound impression of the molar to be capped; it was then covered with heavy tin foil, burnished down, and was set into some more of the same metal poured into one of the little square boxes which the depots send our right angle burs in. In this way a die and counter sufficient for such use may be made in a few minutes. The tin foil prevents their sticking together. —*Archives.*

THE INFLUENCE OF HEREDITY IN THE CAUSATION OF TUBERCULOSIS OF BONE.—It is a matter of common observation that patients suffering from tuberculosis of the bones and joints, as well as those with the pulmonary form of the disease, not infrequently give a history of tuberculous troubles in the immediate family, especially in one or the other parent.

Some observations recently published by Dr. Julius Dollinger, of Budapest, in the *Centralblatt fur Chirurgie*, No. 35, 1889, tend to confirm the belief in an hereditary predisposition which the general experience of practitioners has established.

Dr. Dollinger found, upon making inquiry of the parents or guardians of the children brought to him on account of bone or joint tuberculosis, that in a large proportion of cases one or more of the immediate ancestors had suffered from pulmonary phthisis. Usually, however, it was not the parents alone who were the sufferers, but the grand-parents also, and not infrequently only the latter. The writer concludes, therefore, that bone phthisis occurs not in the children but in the grandchildren of those who have died from pulmonary tuberculosis. The influence of the tuberculous virus must be exerted through several generations before the normal resistance of the osseous structures is so far weakened that they become a suitable field for the lodgement and development of the tubercle bacillus. In other words, the author's experience adds weight to the common belief that the so-called scrofulous affections are a modified form of tuberculosis, and would seem to justify the retention of the term scrofula as expressing a mild, or at least a modified, form of the tuberculous disease.—*Medical Record.*

---

GOODNESS OF SEEDS.—One lady writes: "I thank you for the goodness of your seeds, which can be honestly recommended and depended upon." Another, "We don't recollect having been without your seeds since we put our hands to amateur gardening, because we have never found any that excelled or even approached yours in reliability. For the last twenty years your seeds have filled our little garden with the choicest flowers." These are some of the reasons why you should send 10 cents to JAMES VICK, Seedsman, Rochester, N. Y., for his new FLORAL GUIDE, which amount may be deducted from the first order. It don't seem possible such a work can be supplied for the money.

---

The following formula is given by the *Chemist and Druggist:*

| | |
|---|---|
| Precipitated chalk - - - | 15 ozs. |
| Soap, powdered - - - - | 1 oz. |
| Saccharin - - - - | 10 grs. |
| Thymol - - - - | 15 grs. |
| Camphor - - - - | 30 grs. |
| Vanillin - - - - - | 5 grs. |
| Oil of rose - - - - | 6 drops. |

Rub the camphor and thymol together in a mortar, and warm gently so as to render the mixture liquid; then add the chalk in small portions at a time, reserving about one ounce; next add the other ingredients, the perfumes being first separately rubbed with the remainder of the chalk.—*Druggists' Circular*, Feb. 1880.

---

A POPULAR BELIEF IN THE CONTAGIOUSNESS OF PHTHISIS—In a paper on the contagiousness of pulmonary phthisis, read at the twenty-fifth anniversary meeting of the Caucasian Medical Society, Dr. Babayeff mentioned the curious fact that among the Georgians the name for consumption is "chlekki," meaning "the contagious disease." When one of their number is found to be suffering from this disease he is at once isolated, and is taken to a hut or tent at some distance from the village. The care of these patients is entrusted to an old woman who carries to them the necessary food and drink, and they are never allowed to associate with the well.—*Medical Record.*

